# Inflammation in Diabetic Nephropathy

**DOI:** 10.1155/2012/146154

**Published:** 2012-08-21

**Authors:** Andy K. H. Lim, Gregory H. Tesch

**Affiliations:** ^1^Department of Nephrology, Monash Medical Centre, 246 Clayton Road, Clayton, VIC 3168, Australia; ^2^Monash University Department of Medicine, Monash Medical Centre, 246 Clayton Road, Clayton, VIC 3168, Australia

## Abstract

Diabetic nephropathy is the leading cause of end-stage kidney disease worldwide but current treatments remain suboptimal. This review examines the evidence for inflammation in the development and progression of diabetic nephropathy in both experimental and human diabetes, and provides an update on recent novel experimental approaches targeting inflammation and the lessons we have learned from these approaches. We highlight the important role of inflammatory cells in the kidney, particularly infiltrating macrophages, T-lymphocytes and the subpopulation of regulatory T cells. The possible link between immune deposition and diabetic nephropathy is explored, along with the recently described immune complexes of anti-oxidized low-density lipoproteins. We also briefly discuss some of the major inflammatory cytokines involved in the pathogenesis of diabetic nephropathy, including the role of adipokines. Lastly, we present the latest data on the pathogenic role of the stress-activated protein kinases in diabetic nephropathy, from studies on the p38 mitogen activated protein kinase and the c-Jun amino terminal kinase cell signalling pathways. The genetic and pharmacological approaches which reduce inflammation in diabetic nephropathy have not only enhanced our understanding of the pathophysiology of the disease but shown promise as potential therapeutic strategies.

## 1. Introduction

Diabetic nephropathy (DN) has not been traditionally considered an inflammatory disease. However, recent studies have shown that kidney inflammation is crucial in promoting the development and progression of DN. Inflammation may be a key factor which is activated by the metabolic, biochemical, and haemodynamic derangements known to exist in the diabetic kidney. In this paper, we discuss the evidence for inflammation in DN and the lessons we have learned from novel experimental anti-inflammatory therapies. The main areas covered include the role of immune and inflammatory cells, inflammatory cytokines, and stress-activated protein kinases. We also briefly review the controversy around the role of immune complexes and immune deposition in DN.

## 2. Inflammatory Cells

In human DN, macrophages and T cells accumulate in the glomeruli and interstitium, even in the early stages of the disease. Recruitment of leukocytes involves three steps: (a) selectin-dependent leukocyte rolling on the endothelium, (b) chemokine-dependent integrin activation and leukocyte adhesion, and (c) transmigration of leukocytes across the endothelium [1]. Proinflammatory cytokines produced by leukocytes such as interleukin-1 (IL-1), tumour necrosis factor-*α* (TNF-*α*), and interferon-*γ* (INF-*γ*) can induce resident renal cells to produce a spectrum of chemokines. Elements of the diabetic milieu such as high glucose and advanced glycation end products (AGEs) are also potent stimulators of chemokine production. These chemokines include interleukin-8 (CXCL8), monocyte-chemoattractant protein-1 (MCP-1), INF-*γ* inducible protein (CXCL10), macrophage inflammatory protein-1*α* (MIP-1*α*/CCL3), and RANTES (CCL5). The elaborated chemokines then further direct the migration of additional leukocytes into the kidney and set up an inflammatory cycle. 

### 2.1. Macrophages

Macrophages are key inflammatory cells mediating kidney inflammation in experimental and human diabetes. Activated macrophages elaborate a host of proinflammatory, profibrotic, and antiangiogenic factors. These macrophage-derived products include but are not limited to TNF-*α*, IL-1, IL-6, reactive oxygen species (ROS), plasminogen activator inhibitor-1 (PAI-1), matrix metalloproteinases, transforming growth factor-*β* (TGF-*β*), platelet-derived growth factor (PDGF), angiotensin II, and endothelin [1]. In experimental diabetic mice, macrophage accumulation and activation are associated with prolonged hyperglycaemia, glomerular immune complex deposition, increased chemokine production, and progressive fibrosis [2, 3]. In human type 2 diabetes, kidney macrophage accumulation is associated with the degree of glomerular sclerosis [4]. In another human study, interstitial macrophage accumulation correlated strongly with serum creatinine, proteinuria, and interstitial fibrosis at the time of biopsy, and inversely with the renal function decline (slope of 1/serum creatinine) over the following 5 years [5]. These human data support animal studies in suggesting a pathological role for macrophages in DN.

Strategies impairing kidney leukocyte recruitment have added evidence that macrophages mediate diabetic kidney injury. Increased kidney expression of intercellular adhesion molecule-1 (ICAM-1) has been noted in models of type 1 and type 2 diabetes. ICAM-1 serves as a ligand for LFA-1 on monocytes, facilitating leukocyte adhesion and transmigration. Diabetic ICAM-1 knockout mice showed significant reduction in albuminuria, glomerular, and tubulointerstitial injury, in association with a reduction in macrophage accumulation in the kidney [6]. However, ICAM-1 deficiency affects macrophages and lymphocytes, so these studies do not distinguish the roles of macrophages and lymphocytes. 

Like ICAM-1, MCP-1 is also significantly increased in DN and expression levels correlate with the number of infiltrating interstitial macrophages. Studies suggest that renal MCP-1 is involved in the direction of macrophage migration into the diabetic kidney, while proteinuria itself may contribute to this upregulation of MCP-1 [7]. Blockade of the MCP-1 receptor (CCR-2) with a selective antagonist ameliorated diabetic glomerular sclerosis [8]. Similarly, diabetic *db/db* mice and streptozotocin-(STZ-) induced diabetic mice which are deficient in the gene encoding MCP-1 (*Ccl2*) are protected from renal injury [9, 10]. To further test the pathogenic role of macrophages, we used *c-fms* blockade to selectively target macrophages during the progression of experimental DN [11]. *c-fms* is the receptor for colony stimulating factor-1 (CSF-1), the major cytokine promoting macrophage accumulation, activation, and survival. We administered a neutralizing anti-*c-fms* monoclonal antibody to diabetic *db/db* mice with established albuminuria. This inhibition suppressed inflammation in the diabetic kidney, as evidenced by the reduction in macrophage accumulation, activation, and proliferation. Along with the effects on macrophages, there was a reduction in MCP-1, TNF-*α*, and c-Jun amino terminal kinase (JNK) activation. This resulted in a reduction in glomerular hyperfiltration, tubulointerstitial injury, and fibrosis. 

The evidence so far indicates that infiltrating macrophages are associated with chronic, low-grade inflammation. Macrophages can interact with resident renal cells to generate a proinflammatory microenvironment that amplifies tissue injury and promotes scarring. As we have shown, macrophage-mediated injury is amenable to novel secondary prevention strategies.

### 2.2. Lymphocytes

A T cell infiltrate into diabetic kidneys has long been appreciated but not well studied. The role of T cells in kidney disease is better characterized in crescentic glomerulonephritis, such as antiglomerular basement membrane (GBM) disease [12]. Kidney infiltration by CD4^+^ and CD8^+^ T cells has been noted in diabetic *db/db* mice and diabetic NOD mice [2, 13]. In the latter study, glomerular B-cells were also found to be increased. A kidney T cell influx is common among young patients with early type 1 diabetes, especially those with a shorter duration of diabetes, and correlates with renal function and albuminuria [14]. This T cell accumulation has been noted in the juxtaglomerular apparatus of these patients but the functional role of T cells in this compartment is unclear.

It has been reported that the homing of Th1 cells in glomeruli is P-selectin and ICAM-1 dependent and associated with increased levels of IFN-*γ* and macrophage migration inhibitory factor (MIF) in crescentic Th1-mediated glomerulonephritis [15]. Although the mechanisms of Th1 cell migration in models of DN have not been reported yet, levels of ICAM-1 and P-selectin are increased within the diabetic kidney. As T cells constitutively express LFA-1, and ICAM-1 expression is found on renal endothelial, epithelial, and mesangial cells, it is likely that this interaction plays a significant role in T cell migration into the kidney. This is supported by a study showing that glomerular accumulation of CD4^+^ T cells was decreased in ICAM-1-deficient *db/db* mice [6]. 

Human and rat T cells also express receptors for AGEs. The activation of CD4^+^ and CD8^+^ T cells by AGEs can initiate INF-*γ* secretion by T cells, which will induce further inflammation and oxidative stress within renal tissues [16]. T cells also have the capacity to recruit and activate macrophages through Th1-driven INF-*γ* production. However, the functional role of T cells varies depending on the model studied. For example, *α*3 (IV) collagen/*RAG1* double-knockout mice were not protected from the development of glomerulonephritis but were protected from interstitial fibrosis [17]. On the other hand, inhibition of regulatory T cells (Tregs) with anti-CD25 monoclonal antibody worsened renal injury in models of ischaemia-reperfusion [18]. 

To further explore the role of lymphocytes, we studied the kidney outcomes in recombination activating gene-1- (*RAG1-*) deficient mice made diabetic with STZ injections [19]. *RAG1*-deficient mice lack mature T and B cells and thus lymphocytes are not recruited into the diabetic kidney. *RAG1*-deficient mice were not protected from histological injury, renal fibrosis, or reduced creatinine clearance. However, *RAG*1^−/−^ diabetic mice showed significant attenuation of albuminuria, associated with preservation of podocytes, and a reduction in glomerular macrophage activation. This would suggest that lymphocytes had a lesser role in inflammation but were involved in the pathogenesis of albuminuria. The glomeruli of *RAG*1^−/−^ diabetic mice were also devoid of immunoglobulin staining, in contrast to their *RAG1 *intact counterparts. This led us to consider the possible role of B cells and immunoglobulin deposition as a potential contributor to glomerular inflammation and albuminuria (see next section).

One subset of T cells which has raised intense interest is the Forkhead box P3 (CD4^+^CD25^+^Foxp3^+^) regulatory T cell (or Treg). These cells may participate in dampening the inflammation in the diabetic kidney, given the worsening of renal injury when Tregs are inhibited in ischaemia-reperfusion models. STZ-diabetic mice have increased peripheral Treg in the circulation, spleen, and lymph nodes. However, these cells appear to be dysfunctional [20]. Tregs are also increased in the kidneys of STZ-diabetic mice [19]. When diabetic *db/db* mice were depleted of Tregs, they demonstrated enhanced kidney inflammation, leading to worsening albuminuria and glomerular hyperfiltration. Furthermore, adoptive transfer of Tregs significantly improved insulin sensitivity and reduced nephropathy in the *db/db* mice [21]. These findings suggest that the manipulation of Treg number or function may be useful in attenuating inflammation in DN.

### 2.3. Immune Deposition

A number of modified proteins which develop in diabetes are potentially immunogenic. This includes human immune responses to oxidized low-density lipoproteins (LDL), which may subsequently result in the formation of antioxidized LDL immune complexes [22, 23]. In patients with type 1 diabetes, these immune complexes are associated with macroalbuminuria [24]. Another study of type 1 diabetes demonstrated significant positive correlations between IgG antibody concentration isolated from circulating immune complexes and the serum creatinine and albumin excretion [25]. These antibodies were mostly of the proinflammatory IgG1 and IgG3 isotypes. IgG antibodies were proportionally greater than IgM by a ratio of 8 : 1. These immune complexes have been shown *in vitro* to stimulate production of MCP-1 and CSF-1 [26], and promote glomerular fibrosis by stimulating collagen production by mesangial cells [27]. Oxidized LDL immune complexes are also capable of activating the classical pathway of complement and inducing proinflammatory cytokine production by human macrophages, including IL-1, IL-6, and TNF-*α* [28]. These responses occur through the ligation of Fc*γ* receptors on mesangial cells and macrophages and may involve the activation of the p38 mitogen-activated protein kinase (p38 MAPK), JNK, and protein kinase C (PKC) pathways [27].

Circulating immune complexes and glomerular IgG deposition have long been recognized in diabetic rodent models [13, 29]. Similarly, immune deposits have also been described in histological studies of DN [30]. In an analysis of 567 kidney biopsies from patients with type 1 and 2 diabetes, approximately 30% of glomerular disease present was immune complex and secondary focal glomerulosclerosis [31]. This does raise a valid question as to the role of these immune complexes in the pathogenesis of DN. However, these immune deposition and immune complexes have usually been dismissed as concurrent or unrelated diseases. This is reflected by the stance of the Animal Models of Diabetic Complications Consortium (AMDCC) on excluding immune deposition in models of DN [32]. It may require a major paradigm shift before researchers are able to finally lay this issue to rest. Our study of *RAG1*-deficient mice suggests the possible involvement of immune deposition in promoting albuminuria. We are currently undertaking further studies in this area.

## 3. Inflammatory Cytokines

As our knowledge of DN expands, a number of inflammatory cytokines have emerged as being closely involved in the pathogenesis of DN. Some of the major inflammatory cytokines which are believed to play an important role in DN are discussed here (summarised in Table 1). The role of cytokines and growth factors in diabetic kidney disease has also been specifically reviewed elsewhere [33, 34]. 

### 3.1. TNF-*α*

TNF-*α* is mainly produced by monocytes, macrophages, and T cells. However, resident renal cells are also able to produce TNF-*α*, including mesangial, glomerular, endothelial, dendritic, and renal tubular cells [35–38]. TNF-*α* expression is increased in the kidneys of experimental diabetic rats [39]. The effects of TNF-*α* include promotion of local reactive oxygen species (ROS) generation [40–42], increasing albumin permeability [42], and the induction of cytotoxicity, apoptosis, and necrosis [43, 44]. TNF-*α* is implicated in the recruitment of monocyte-macrophages, reducing glomerular filtration rate (GFR) by haemodynamic changes [45–48], as well as altering endothelial permeability [49]. In line with experimental data, patients with type 2 diabetes have 3-4 times greater serum levels of TNF-*α* compared to nondiabetic patients, and these levels are higher in diabetic patients with microalbuminuria compared with those that have normoalbuminuria [50, 51]. Similarly, urinary TNF-*α* excretion correlates well with the clinical markers of DN and progression of disease [52].

### 3.2. MCP-1

MCP-1 promotes monocyte and macrophage migration and activation, upregulates expression of adhesion molecules, and promotes the expression of other proinflammatory cytokines [53, 54]. MCP-1 increases progressively in diabetic kidneys in animals models [2, 55]. It is produced by various cells in the kidney, including monocyte-macrophages, mesangial cells, podocytes, and tubular cells [10, 55, 56]. Recent studies have convincingly demonstrated the causative role of MCP-1 in experimental DN. In models of type 1 and 2 diabetes, renal injury was attenuated in MCP-1-deficient animals [9, 10]. Patients with type 2 diabetes and nephropathy excrete high levels of MCP-1 in the urine, which correlates with albuminuria and N-acetyl-*β*-D-glucosaminidase (NAG) excretion as a marker of tubular injury [7]. Interestingly, inhibition of ACE or the mineralocorticoid receptor also suppresses renal MCP-1 production [57, 58]. However, it remains to be determined if direct inhibition of MCP-1 will be more effective.

### 3.3. ICAM-1

ICAM-1 is a cell surface glycoprotein involved in leukocyte attachment to the endothelium as the ligand for LFA-1(integrin) on leukocytes. ICAM-1 is also present on the membranes of macrophages and lymphocytes. ICAM-1 expression is increased in models of type 1 and 2 diabetes [59, 60]. It can be induced by hyperglycaemia, AGEs, oxidative stress, hyperlipidaemia, hyperinsulinaemia, and proinflammatory cytokines [1]. Recent studies have demonstrated that mice deficient in ICAM-1 are protected against macrophage accumulation and nephropathy in models of type 1 and 2 diabetes [6, 61]. A soluble form of ICAM-1 has been described as increased in patients with type 2 diabetes and DN [62]. However, there is a lack of data from human studies on the role of ICAM-1 in DN. 

### 3.4. VCAM-1

Vascular cell adhesion molecule-1 (VCAM-1) is another molecule involved in leukocyte-endothelial adhesion, which facilitates leukocyte recruitment into the kidney during inflammation. VCAM-1 expression is increased in the kidneys of patients with DN [63] and in diabetic rodents [64, 65]. During diabetes, VCAM-1 expression is detected on vascular endothelium and infiltrating cells in the kidney [64]. Increasing plasma levels of soluble VCAM-1 are associated with the progression of albuminuria in patients with type 1 and type 2 diabetes [66, 67], suggesting that sVCAM-1 may be a useful biomarker of diabetic renal injury. However, it is unclear whether circulating sVCAM-1 levels correlate with inflammation in diabetic kidneys.

### 3.5. Interleukin-1

Increased expression of IL-1 is found in experimental DN [68, 69]. IL-1 is able to enhance ICAM-1, (VCAM-1), and E-cadherin expression [70, 71]. Furthermore, IL-1 is able to induce endothelial cell permeability, alter glomerular haemodynamics by affecting prostaglandin synthesis, stimulate mesangial and fibroblast proliferation, and induce TGF-*β*1 production [72–74]. Polymorphisms of the IL-1  *β* and IL-1 receptor genes were found to be associated with an increased risk of end-stage kidney disease in Korean patients with type 2 diabetes [75]. However, polymorphisms in the IL-1 gene cluster were not found to contribute to the genetic susceptibility of DN in Caucasian patients with type 1 diabetes [76]. Further investigation is required to determine the importance of IL-1 in human DN.

### 3.6. Interleukin-6

IL-6 is produced by endothelial cells, leukocytes, adipocytes, and mesangial cells. Experimental studies have shown IL-6 overexpression in diabetic kidneys, which correlate with kidney hypertrophy and albumin excretion [68, 77]. IL-6 has been suggested to mediate endothelial permeability, mesangial proliferation, and increased fibronectin expression [78–81]. IL-6 is increased in patients with type 1 and 2 diabetes with DN, and IL-6 levels are higher in patients with overt proteinuria compared to microalbuminuria or normoalbuminuria [82, 83]. Increased IL-6 is also associated with GBM thickening in type 2 diabetes patients and mesangial expansion in kidney biopsies of diabetic patients [79, 84].

### 3.7. Interleukin-18

IL-18 is a potent inflammatory cytokine that induces IFN-*γ* [85] and the production of other proinflammatory cytokines (IL-1 and TNF-*α*), upregulation of ICAM-1, as well as apoptosis of endothelial cells [86–88]. Tubular epithelial cells are the major source of IL-18 but recent studies have also demonstrated IL-18 production from infiltrating monocyte-macrophages and T cells [89, 90]. Serum and urinary IL-18 are increased in type 2 diabetes patients and correlate with urinary albumin excretion [50, 91].

### 3.8. Adipokines

Adiponectin, leptin, and resistin are cytokines produced by adipose tissue. Adiponectin regulates insulin sensitivity, and also has anti-inflammatory and antioxidant properties. Adiponectin suppresses TNF-*α*-induced upregulation of endothelial cell adhesion molecules and interferes with leukocyte rolling and adhesion [92]. Adiponectin also suppresses leukocyte colony formation and reduces TNF-*α* secretion by macrophages [93, 94]. Adiponectin may also interfere with receptor activation for platelet-derived growth factor (PDGF), fibroblast growth factor (FGF), and epidermal growth factor (EGF) [95]. In diabetic *db/db* mice, ezetimibe treatment normalized adiponectin levels and enhanced kidney expression of adiponectin receptor 1 [96]. With this, there was a 50% reduction in albuminuria and improved glomerular hypertrophy. STZ-induced diabetic rats overexpressing adiponectin showed improvements in markers of endothelial function, including lower levels of endothelin-1, plasminogen activator inhibitor-1, and inducible nitric oxide synthase [97]. This was associated with preservation of nephrin, lower TGF-*β* levels and a reduction in proteinuria. In human type 1 and 2 diabetes, serum adiponectin levels are already elevated and positively correlated with both albuminuria and serum creatinine [98–100]. Thus, it is unclear if further manipulation of adiponectin levels would be beneficial in humans. 

In contrast to adiponectin, leptin exerts proinflammatory effects. Experimentally, these effects include stimulation of inflammatory signalling pathways and oxidative stress, impairment of endothelial function and platelet aggregation, and hypertrophy and proliferation of vascular smooth muscle cells [101]. The data in humans is less clear. In type 2 diabetes, both low and high serum leptin levels were risk factors for declining renal function. Furthermore, lower serum leptin levels were associated with progression of albuminuria [102]. Another study noted micro- and macroalbuminuria patients with type 2 diabetes had higher leptin levels than normoalbuminuric patients [103]. However, leptin levels may also be elevated in end-stage kidney disease due to reduced degradation.

Resistin promotes expression of endothelin-1, VCAM-1, and MCP-1. Resistin also promotes proliferation of vascular smooth muscle cells via extracellular regulated kinase (ERK) and Akt signalling pathways and inhibits insulin signalling and endothelial nitric oxide synthase (eNOS) activation [101]. The role of resistin in human DN is unclear. In type 2 diabetes patients, resistin was elevated in patients receiving loop diuretics but not thiazides. Serum resistin levels also increase in advanced CKD [104].

## 4. Stress-Activated Protein Kinases (SAPKs)

p38 MAPK and JNK are stress-activated protein kinases (SAPKs). Receptor activation by various stress stimuli on the cell surface trigger intracellular signalling involving a cascade of phosphorylation by MAP kinase kinase kinase (MAP3K), a MAP kinase kinase (MAP2K), and finally the MAPK/SAPK [105]. There is overlap and redundancy in the ability of different MAP3K's to activate p38 MAPK or JNK. However, direct activation of p38 MAPK and JNK can only occur through MKK3/6 and MKK4/7, respectively. 

### 4.1. p38 MAPK

p38 MAPK has four isoforms (p38*α*, *β*, *γ*, and *δ*) which are all expressed by kidney cells. However, activation of p38*α* is most strongly associated with renal inflammation and injury. Recent clinical studies have demonstrated that kidney p38 MAPK activity is increased and associated with the development of DN [106, 107]. Renal biopsies from patients with established type 2 diabetes display prominent glomerular and tubulointerstitial p38 MAPK signalling despite treatment with angiotensin inhibitors [106]. In diabetic animal models, p38 MAPK activity rapidly increases in glomeruli and tubules after the induction of hyperglycaemia, and is also found in the accumulating kidney interstitial cells associated with advanced nephropathy. Studies of nondiabetic kidney disease have shown that pharmacological inhibition of p38 MAPK suppressed inflammation and fibrosis [108, 109].

*In vitro* studies have identified specific kidney cells and mechanisms of renal injury that may be affected by p38 MAPK signalling during diabetes. Exposure to high glucose activates p38 MAPK in human mesangial cells [110], mouse podocytes [111], and rat proximal tubular cells [112]. Similarly, glycated albumin can stimulate p38 MAPK phosphorylation in cultured fibroblasts [113]. Activation of p38 MAPK has been shown to promote apoptosis of rat mesangial cells exposed to methylglyoxal [114] and apoptosis of mouse podocytes following stimulation with TGF-*β* [115] and AGEs [116, 117]. In addition, p38 MAPK signalling can contribute to proinflammatory and profibrotic responses. p38 MAPK activation enhances production of MCP-1 by vascular endothelial cells [118], induces local angiotensinogen production in rat tubular cells [112], stimulates TGF-*β*-induced fibronectin accumulation in renal interstitial fibroblasts [119] and collagen production in mouse mesangial cells [120], increases TGF-*β* expression in renal tubular cells [121], and promotes synthesis of vascular endothelial growth factor (VEGF) induced by angiotensin II [122, 123]. Studies have also shown that p38 MAPK signalling mediates high-glucose-induced tubular hypertrophy [121] and transactivation of the epidermal growth factor receptor required for dedifferentiation of proximal tubular epithelial cells following oxidant injury [124]. 

Functional blocking studies are required to determine the pathogenic role of p38 MAPK signalling in diabetes and its complications. Inhibitors of p38*α* and p38*β* have been used in a number of nondiabetic models to reduce proteinuria and inflammation [105]. In rats with STZ-induced diabetes, a p38 MAPK inhibitor (FR167653) ameliorated the increased glomerular fibronectin mRNA and protein and reduced mesangial cell apoptosis. Similar results were obtained in high-glucose stimulated cultured rat mesangial cells [125]. However, the investigators did not study the effects of p38 MAPK inhibition on kidney inflammatory cells, albumin excretion, tubulointerstitial injury, or renal function. 

We utilized a genetic approach to studying the p38 MAPK pathway in DN. As genetic deletion of p38*α* is lethal, our strategy was to target the immediate upstream kinases that regulate p38 MAPK signalling. The MKK3 and MKK6 kinases provide a parallel and independent mechanism of phosphorylating p38 MAPK but their relative contribution to the increased p38 MAPK activity associated with DN was unknown. MKK3 appeared to be the most attractive target because MKK3-p38 MAPK signalling has been shown to be nonredundant in some pathological processes *in vitro* [126, 127]. We studied the effects of MKK3-p38 MAPK inhibition on kidney outcomes in *Mkk3*-gene-deficient diabetic *db/db* mice [128]. In the absence of MKK3 signalling, we noted an attenuation of *Ccl2* expression (hence MCP-1 levels) and interstitial macrophage accumulation. The result was a reduction in albuminuria with concomitant podocyte preservation and reduced mesangial cell activation. Glomerular sclerosis and tubulointerstitial injury were also attenuated. This study demonstrated a pathogenic role of the MKK3-p38 signalling pathway in the progression of DN and may be a viable target for intervention. There are also nonredundant functions of the upstream kinases which confirm previous *in vitro* findings.

### 4.2. JNK

There are three main JNK isoforms. JNK1 and JNK2 are expressed in the kidney but JNK3 is limited to the nervous system [129]. Phosphorylated JNK translocates into the nucleus and activates transcription factors and cellular responses such as inflammation or apoptosis. For example, *in vitro* studies have demonstrated that inhibition of JNK ameliorates the induction of apoptosis by oxidative stress in tubular epithelial cells [130]. A unique JNK target is the phosphorylation of Ser63 and Ser73 in the NH_2_-terminal domain of c-Jun, which can be used as a surrogate marker of JNK activity [131]. JNK-dependent signalling is important in normal development because genetic deficiency of both JNK1 and JNK2 are foetal lethal. JNK can be activated by various elements of the diabetic milieu, including hyperglycaemia, AGEs, angiotensin II, ROS, and proinflammatory cytokines (IL-1, TNF-*α*) [132]. 

JNK may be important in CSF-1 signalling through *c-fms*, thus promoting monocyte-macrophage differentiation, development, survival, and function [133, 134]. In animal models of anti-GBM disease and unilateral ureteric obstruction, treatment with the JNK inhibitor CC-401 reduced renal injury through modulation of macrophage activation [135, 136]. In biopsy samples of human DN, JNK activation correlated with interstitial macrophage accumulation, kidney injury molecule-1 (KIM-1) expression, interstitial fibrosis, and loss of renal function [137, 138]. 

We examined the effects of JNK blockade in a STZ model of diabetes in spontaneously hypertensive rats by administering a JNK inhibitor (CC-930) at the onset of detectable kidney JNK activation (phosphorylated-JNK) and albuminuria [139]. JNK inhibition resulted in a reduction in macrophage accumulation and *Ccl2* mRNA (encoding MCP-1). However, JNK inhibition exacerbated albuminuria in association with accelerated loss of glomerular nephrin and podocin. Similar negative outcomes were reported in *db/db* mice treated with a TAT-JNK inhibitor peptide, which exacerbated albuminuria and nephrin loss despite improvements in insulin sensitivity [140]. Together, these studies demonstrate that blockade of JNK signalling causes significant injury to podocytes while also suppressing kidney inflammation in animal models of early DN. Whether the benefits of JNK inhibition outweigh its effects on podocyte damage in more advanced stages of diabetic renal injury remains to be determined.

### 4.3. Kidney Inflammation in Type 1 versus Type 2 Diabetic Nephropathy

Analysis of renal biopsies from type 1 and type 2 diabetic patients who develop DN indicates that the inflammatory infiltrate is similar in both groups [5], which is consistent with studies in diabetic animal models [2, 3]. In both type 1 and type 2 models, kidney inflammation in diabetic rodents correlates strongly with the development of hyperglycaemia and glycated haemoglobin, and is driven by an increased kidney production of chemokines and proinflammatory cytokines [2, 3], and induction of kidney SAPK signalling [106]. In addition, the coexistence of hypertension or hyperlipidaemia exacerbates kidney inflammation in both type 1 and type 2 diabetes [137, 141–144].

## 5. Conclusion

**Figure 1:**
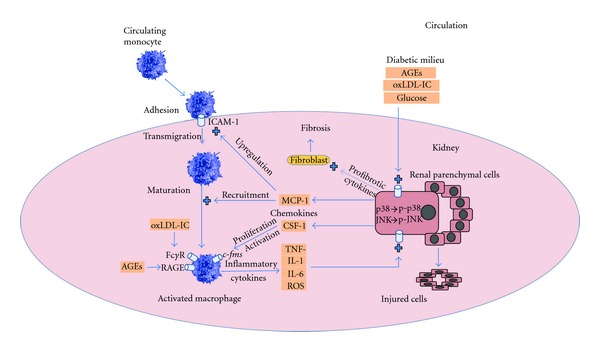
The inflammatory amplification loop in the diabetic kidney. Circulating immune cells such as monocytes are recruited into the diabetic kidney due to upregulation of adhesion molecules such as ICAM-1. Chemokines such as MCP-1 act as chemoattractants which promote accumulation of the immune cells in the kidney. These immune cells are activated by numerous signals such as the ligation of *c-fms* by CSF-1, receptor for AGE by AGEs, and the Fc*γ* receptors by antioxidized LDL immune complexes. CSF-1 also promotes the maturation, proliferation, and survival of monocyte-macrophages. Activated immune cells act as inflammatory cells and elaborate proinflammatory cytokines and reactive oxygen species (ROS), which trigger a cell signalling cascade mediated by the stress-activated protein kinases, p38 MAPK, and JNK. These kidney cells then respond by the production of chemokines such as MCP-1 and CSF-1, and profibrotic factors such as TGF-*β* which increase extracellular matrix production by mesangial cells and interstitial fibroblasts. Ultimately, there is cellular injury and progressive fibrosis within the diabetic kidney.

Inflammation plays an essential role in the progression of DN. Recent evidence indicates that innate immunity, rather than adaptive immunity, is the major driving factor in the inflammatory response in diabetic kidneys. The main components of this immune response (infiltrating cell types, cytokines, signalling pathways) are described in this paper (summarized in Figure 1). Our current knowledge indicates that elements of the diabetic milieu (hyperglycaemia, AGEs, immune complexes) can activate kidney cells via induction of SAPK signalling, resulting in the release of chemokines and upregulation of cell adhesion molecules. These events facilitate the kidney infiltration of monocytes and lymphocytes, which become activated in the diabetic kidney and secrete injurious molecules, such as proinflammatory cytokines and reactive oxygen species. This leukocyte activity amplifies the inflammatory response and promotes cell injury and the development of fibrosis. Better understanding of the inflammatory response in diabetic kidneys is expected to identify novel anti-inflammatory strategies for the potential treatment of human DN.

## Figures and Tables

**Table 1:** Cytokines involved in Diabetic Kidney Inflammation.

Cytokine	Role in Diabetic Kidney Inflammation
ICAM-1	Adhesion molecule facilitating leukocyte-endothelial adhesion and infiltration into diabetic kidneys
VCAM-1	Adhesion molecule facilitating leukocyte-endothelial adhesion and infiltration into diabetic kidneys
MCP-1	Chemoattractant which promotes macrophage recruitment into diabetic kidneys
TNF-*α*	Promotes production of reactive oxygen species, induces cell injury, and increases endothelial permeability
IL-1	Stimulates expression of cell adhesion molecules and profibrotic growth factors and increases endothelial permeability
IL-6	Promotes mesangial proliferation, glomerular hypertrophy, fibronectin production and increases endothelial permeability
IL-18	Increases production of other cytokines (ICAM-1, IL-1, TNF-*α*) and induces apoptosis of endothelial cells
Adiponectin	Reduces oxidative stress, production of TNF-*α*, and leukocyte-endothelial adhesion
Leptin	Induces oxidative stress, inflammation, hypertrophy, and proliferation of vascular smooth muscle cells, and impairs endothelial function
Resistin	Promotes expression of MCP-1, VCAM-1, endothelin-1, and proliferation of vascular smooth muscle cells

## Abbreviations

AGEs: Advanced glycation end products
CSF-1: Colony stimulating factor-1
DN: Diabetic nephropathy
ERK: Extracellular cell regulated kinase
GBM: Glomerular basement membrane
ICAM-1: Intercellular cell adhesion molecule-1
IL: Interleukin
INF-*α*: Interferon-*α*
JNK: c-Jun amino-terminal kinase
MAPK: Mitogen-activated protein kinase
MCP-1: Monocyte chemoattractant protein-1
SAPK: Stress-activated protein kinase
STZ: Streptozotocin
TGF-*β*: Transforming growth factor-*β*
TNF-*α*: Tumour necrosis factor-*α*
VCAM-1: Vascular cell adhesion molecule-1.
